# Clinical and hemodynamic evaluation of schistosomiasis-associated pulmonary arterial hypertension from Egyptian pulmonary hypertension centers: epidemiology, risk factors, and survival determinants

**DOI:** 10.1038/s41598-026-41412-7

**Published:** 2026-03-21

**Authors:** Youssef M. Amin Soliman, Mohamed El-Kassas, Ahmed Abd ElAziz, Mohamed Shaaban Mousa, Mohamed Kamal Hasswa, Sally Magdy, Reem Elkorashy

**Affiliations:** 1 Department of Pulmonary Medicine, Faculty of Medicine, Cairo University, Cairo, Egypt; 2 Department of Endemic Medicine, Faculty of Medicine, Helwan University, Cairo, Egypt; 3 Department of Interventional Cardiology, National Heart Institute, Giza, Egypt

**Keywords:** Bilharzial pulmonary hypertension, Pulmonary hypertension, Schistosomiasis pulmonary hypertension, Cardiology, Diseases, Medical research, Risk factors

## Abstract

Schistosomiasis-associated PAH (Sch-PAH) falls under group I pulmonary hypertension and it affects 230 million people, mainly in sub-Saharan Africa. This significant burden has prompted studies on the clinical characteristics and outcomes of Sch-PAH to improve awareness and management, particularly in developing regions. This study investigates the characteristics of Sch-PAH, including demographics, clinical and hemodynamic features, and survival outcomes compared to idiopathic Pulmonary arterial hypertension (IPAH), while evaluating the prognosis and current clinical practices. A cohort of 83 patients, including 41 with Sch-PAH and 42 with IPAH, were studied retrospectively over five years (2019–2024) from 3 pulmonary hypertension centres in Egypt. Data collection focused on demographic details, comorbidities, echocardiographic findings, and survival rates. Sch-PAH patients were significantly older (50.4 ± 12 years) and predominantly male (*P* < 0.001) compared to IPAH patients (34.5 ± 9.7 years). Higher comorbidity rates, chiefly chest and hepatic disorders, were observed in Sch-PAH. The Sch-PAH group exhibited more WHO functional class IV cases and greater left atrium (LA) and pulmonary artery (PA) dilation (*P* = 0.007, *P* = 0.006, *P* = 0.01, respectively). Lower ejection fraction (EF%) and absence of PA dilation and portal hypertension were linked to improved survival, with EF% emerging as an independent survival predictor (OR = 0.72 and 0.55; *P* = 0.002 and < 0.001). The EF% cutoff below 66% showed high predictive accuracy for survival (AUC = 0.85, *P* = 0.008). Sch-PAH affects primarily older males with significant comorbidities. Its prevalence poses a public health challenge, making the identification of mortality predictors crucial for early patient risk assessment and improved prognosis management.

## Introduction

Pulmonary arterial hypertension (PAH) is a severe condition that can arise from various underlying diseases. Classified as group I pulmonary hypertension, it is subdivided into idiopathic pulmonary arterial hypertension (IPAH) and other forms associated with various medical conditions. These include heritable pulmonary arterial hypertension (HPAH), drug- or toxin-induced PAH, congenital heart disease characterized by right-to-left shunts, connective tissue diseases (CTD), as well as infections such as HIV, portal hypertension, and schistosomiasis^1^**.**

Schistosomiasis, particularly its hepatosplenic form, complicates the clinical picture, contributing to PAH in as many as 10% of affected cases^2^ .In this condition, the mean pulmonary artery pressure exceeds 20 mmHg while the pulmonary artery occlusion pressure remains normal (less than 15 mmHg). This situation is typically caused by increased pulmonary vascular resistance exceeding 2 Wood units, which often leads to right-sided heart failure. Schistosomiasis-associated PAH has been recognized as a distinct clinical entity since its initial description in 1932 by S. Azmi Pasha, with the condition being more pronounced in cases involving Schistosoma mansoni compared to Schistosoma haematobium^3^.

Globally, schistosomiasis affects an estimated 230 million individuals across more than 70 countries^4^, with approximately 80% of cases occurring in sub-Saharan Africa^5^. Worldwide, it is considered one of the leading causes of PAH. Notably, the prevalence of schistosomiasis is rising in non-endemic regions, primarily due to increased migration to developed countries^6^.

The significance of schistosomiasis in the context of pulmonary hypertension is particularly critical in certain regions. A recent registry study in Egypt found that schistosomiasis-associated PAH (Sch-PAH) constituted 15.4% of all enrolled PAH cases^7^. Moreover, a study by Farrag et al. in 2012 reported a prevalence of schistosomiasis-associated pulmonary hypertension via echocardiography of 8.6% among seropositive patients in the Delta regions of northern Egypt, an area known for its endemic schistosomiasis^8^. 

In Brazil, a study involving 178 newly diagnosed PAH patients indicated that Sch-PAH accounted for 19.7% of cases^9^.Conversely, another Brazilian study detected pulmonary hypertension in 10.7% of patients with Schistosoma liver fibrosis assessed by transthoracic echocardiography^10^**.**

While the precise mechanisms by which schistosomiasis induces PAH remain largely undefined, it is likely that porto-pulmonary hypertension and inflammation triggered by schistosoma egg antigens play pivotal roles in the development of Sch-PAH^11^.

Increased awareness of the burden associated with various forms of PAH, including schistosomiasis, has spurred research into their clinical-pathological characteristics and patient outcomes. This focus is particularly relevant given the potential eligibility of Sch-PAH for targeted vasodilatory therapies globally^12,13^.

Sch-PAH may be more prevalent, especially in developing nations, underscoring the necessity for heightened awareness regarding its clinical presentation and management^14^.

This study aims to evaluate the demographic, clinical, and hemodynamic characteristics of patients with Sch-PAH compared to those with IPAH, as well as to assess the prognosis and outcomes of Sch-PAH patients within the framework of current clinical practices.

## Methods

This study employed a multicenter retrospective cohort design, collecting data from three pulmonary hypertension centers in Egypt: The Pulmonary Vascular Diseases Unit at Kasr El-Ainy University Hospital, the Pulmonary Hypertension Unit at the National Heart Institute, and the Pulmonary Hypertension Unit at the International Medical Center. The study included patients diagnosed between January 2019 and December 2024, comprising 41 individuals with Sch-PAH and a randomly selected control group of 42 patients with IPAH.

We compared Schistosomiasis-associated PAH (Sch-PAH) with idiopathic pulmonary arterial hypertension (IPAH) as IPAH is the most common form of pulmonary arterial hypertension hence, could be used as control group and both groups are Group I pulmonary hypertension so comparison could be valid.

PAH was defined according to the 2015 guidelines, which were updated in 2022^1^. This definition requires a mean pulmonary artery pressure (mPAP) exceeding 20 mmHg at rest, a pulmonary artery occlusion pressure (PAOP) below 15 mmHg, and a pulmonary vascular resistance (PVR) greater than 2 Wood units, all confirmed via right heart catheterization. Patients were classified as having Sch-PAH if they exhibited liver ultrasonography findings suggestive of schistosomiasis, including peri-portal fibrosis or left lobe enlargement, alongside a history of exposure to or prior treatment for schistosomiasis (Schistosoma species could not be identified as Sch associated PAH usually develops more than 20 years after initial infection).

The collected data included demographic factors such as age, gender, and comorbidities, as well as the WHO functional class, results from the 6-min walk test (6MWT), and echocardiographic parameters. The echocardiographic parameters assessed included pulmonary artery systolic pressure, right atrial and right ventricle sizes, left atrium dilation, and ejection fraction, along with the presence of pericardial effusion. Right heart catheterization parameters measured included mean right atrial pressure (mRAP), mPAP, mixed venous oxygen saturation (SVO2), cardiac index (CI), and PVR. Additionally, other parameters studied included pulmonary artery diameter as measured by chest CT or CT angiography, mortality outcomes, and patient survival duration.

### Informed consent

Written informed consents were collected from all patients prior to participation in this study clarifying the purpose of the study.

Our study followed ethical protocols and methodology according to Helsinki declarations 1964.

### Ethical considerations

The study received approval N-124-2020 from the Institutional Review Board and Ethics Committee of the Faculty of Medicine, Cairo University.

### Statistical methods

Statistical analyses were performed using the Minitab software (version 17.1.0.0). Continuous variables were reported as means with standard deviations, while categorical variables were expressed as counts and percentages. Normality of the data was assessed using the Shapiro-Wilk test. Comparisons between groups were conducted using independent t-tests for continuous data and Chi-square tests for categorical data. Pearson correlation coefficients evaluated linear relationships between variables. Logistic regression analysis identified survival predictors in the Sch-PAH group, adjusting for age, sex, and comorbidity. Prognostic capabilities of ejection fraction were assessed through ROC curve analysis, with significance determined at *P* < 0.05.

## Results

### Population characters

During the five-year period from January 2019 to December 2024, a total of 83 patients were included in the cohort, comprising 41 individuals with Schistosomiasis-Associated Pulmonary Hypertension (Sch-PAH) and 42 individuals with Idiopathic Pulmonary Arterial Hypertension (IPAH), in accordance with the pulmonary hypertension (PH) guidelines from 2015, which were updated in 2022.

The mean age of patients with Sch-PAH was significantly higher at 50.4 ± 12 years compared to 34.5 ± 9.7 years for those with IPAH (*P* < 0.001). Furthermore, there was a notable predominance of male patients in the Sch-PAH group, while the IPAH cohort had a higher percentage of female patients (*P* < 0.001) (Table 1).

**Table 1:** Shows demographic characteristics and hemodynamic data of both groups. Continuous data represented as mean and standard deviation (SD), and categorical data as number and percentage (%).

Parameter	Sch-PAH (n = 41)			IPAH (n = 42)			*P *
**Age (years)** *Gender* Female	Mean/N	SD/%		Mean/N	SD/%		** < 0.001 **^**†**^
	50.4	12		34.45	9.71		
	13	31.71		36	85.71		< 0.001 *
Male **Presence of comorbidity**	28	68.29		6	14.29		
	11	26.83		1	2.38		0.002*
Diabetes mellitus	2	4.88		0	0		0.24*
Cardiovascular disease	3	7.32		1	2.38		0.35*
Chest disease	3	7.32		0	0		0.03*
Hepatic disease (1 HBV, 2HCV, 2HCC) **WHO FC** FC-I	5	12.2		0	0		0.02 *
	2	4.88		3	7.14		0.66*
FC-II	16 39.02			20 47.62			0.42*
FC-III	18		43.9	19		45.24	0.91*
FC-IV **6MWD (meters)** **Echocardiography** PASP (mmHg)	5		12.2	0		0	0.007*
	296.4		82.2	300.5		96	0.83 ^**†**^
	85.1		24.2	85.1		24.2	0.41^**†**^
Dilated RA	39		95.12	37		88.1	0.24*
Dilated RV	39		95.12	39		92.86	0.66*
Dilated LA	18		43.9	7		16.67	0.006*
Pericardial effusion	4		9.76	6		14.29	0.52*
EF (%) **Hemodynamic** mRAP	66.5		6.13	68.11		7.94	0.35 ^**†**^
	9.87		5.98	9.85		5.34	0.98^**†**^
mPAP	54.9		11.8	56.9		16	0.55^**†**^
CI	2.52		1.06	2.56		1.08	0.86^**†**^
PVR	11.91		6.01	15		12.7	0.18^**†**^
Svo2	60.69		8.74	62.6		11.2	0.41^**†**^
**PA dilatation (**by Chest CT > 32 mm) **Duration of illness in** **month** (from the beginning of symptoms till time of diagnosis) **Outcome** Alive All-cause mortality **Mono-Therapy**	6		14.63	0		0	0.01*
	35.4		14.6	28.7		14.9	**0.04**^**†**^
	34 7 5		82.93 17.07 12.2	37 5 1		88.1 11.9 2.38	0.51 0.08*

N, number; SD, standard deviation; †: Independent t-test, *: Chi square test, *P* < 0.05 considered significant.

HBV, hepatitis B virus; HCV, hepatitis C virus; HCC, hepatocellular carcinoma; PA, Pulmonary artery; Sch- PAH, Schistosomiasis-associated pulmonary arterial hypertension; IPHT, Idiopathic pulmonary arterial hypertension; FC, functional class; RA, right atrium; RV, right ventricle; LA, left atrium; EF, ejection fraction; 6MWD, 6 min’ walk distance.

mPAP, mean pulmonary artery pressure; mRAP, mean right atrial pressure; CI, cardiac index; PVR, pulmonary vascular resistance; SVo2, oxygen saturation in the mixed venous blood.

CT, computed tomography.

The frequency of comorbidities was also greater in the Sch-PAH group compared to the IPAH group (*P* = 0.002), with chest and hepatic disorders being the most common comorbidities (*P* = 0.03 and 0.02, respectively). Notably, WHO functional class IV was significantly more prevalent in the Sch-PAH group (*P* = 0.007), while the distribution of the other functional classes was similar between the two groups (Table 1).

Among the Sch-PAH patients, only 10 out of 41 (24.4%) exhibited signs of portal hypertension. In contrast, the remaining 31 patients (75.6%) had a documented history of schistosomiasis and/or had received anti-schistosomal medication, after excluding all other potential causes of PAH.

### Imaging and hemodynamic evaluation

Most echocardiographic and hemodynamic parameters did not show significant differences between the Schistosomiasis-Associated Pulmonary Hypertension (Sch-PAH) group and the Idiopathic Pulmonary Arterial Hypertension (IPAH) group. However, the prevalence of dilated left atrium (LA) was significantly higher in the Sch-PAH group compared to the IPAH group on transthoracic echocardiography (*P* = 0.006). Additionally, pulmonary artery (PA) dilation was more commonly observed in the Sch-PAH group during chest computed tomography (CT) or CT pulmonary angiography, with a mean diameter of 6.12 ± 2.18 cm (*P* = 0.01) (Table 1).

### Medication, risk stratification and outcome of the case

As regards specific pulmonary hypertension medications, monotherapy means using one specific pulmonary hypertension medication as either phosphodiesterase 5 inhibitors (sildenafil, Tadalafil) or endothelin receptor antagonist (Bosentan, Macitentan) while dual therapy or combination therapy means using one drug from phosphodiesterase 5 inhibitors plus another drug from endothelin receptor antagonist.

In our study, we used either sildenafil or Bosentan as monotherapy or both as combination therapy due to affordability in Egypt while other medications are expensive.

**Fig. 1:**
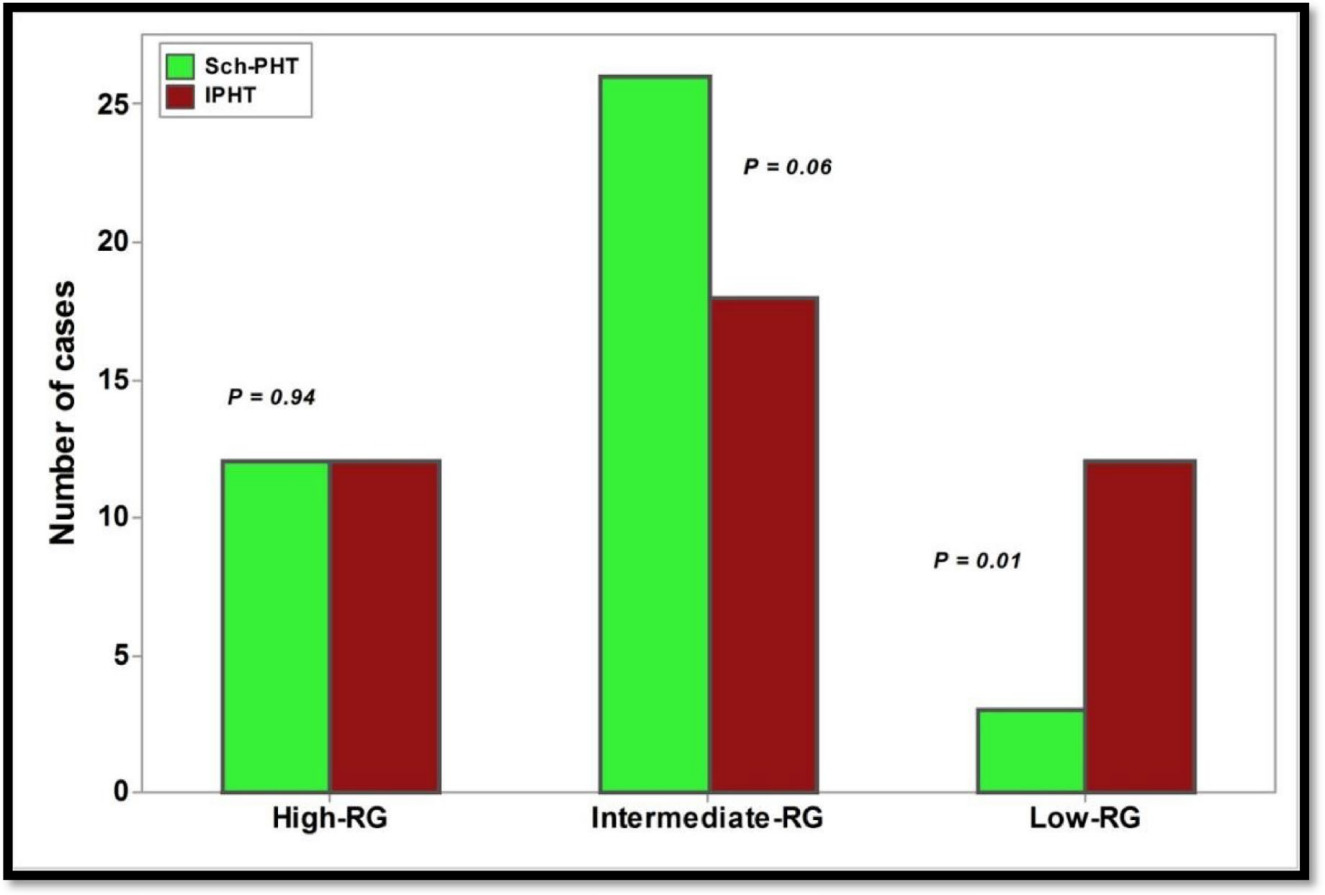
Risk stratification of Sch-PAH and IPAH patients at baseline. Test of significant: Chi square test, *P* < 0.05 considered significant. Figure 1 shows that Sch-PAH patients significantly belong to the high and intermediate risk category, whereas IPAH patients significantly belong to low-risk category, with a *P* value of 0.01. Sch- PAH: Schistosomiasis-associated pulmonary arterial hypertension, IPHT: Idiopathic pulmonary arterial hypertension, RG: Risk group. Risk stratification was identified into low, intermediate or high risk according to pulmonary hypertension guidelines.

The analysis revealed that patients with schistosomiasis-associated pulmonary arterial hypertension (Sch-PAH) predominantly fell into the intermediate and high-risk categories. In contrast, patients with idiopathic pulmonary arterial hypertension (IPAH) were significantly more likely to be classified in the low-risk category (Fig. 1). Risk stratification was identified into low, intermediate or high risk according to multiples parameters as defined by pulmonary hypertension guidelines^1^.

In terms of treatment for Sch-PAH patients, monotherapy was available to 5 out of 41 patients (12.2%), compared to only 1 out of 42 patients (2.4%) in the IPAH group, a difference that was significant (*P* = 0.08) when controlling for both comorbidity and age (Table 1). Additionally, 4 out of the 41 Sch-PAH patients were not receiving specific vasodilator therapies due to financial constraints or personal refusals.

Univariate analysis indicated that lower ejection fraction (EF%) measured by echocardiography was significantly associated with survival (*P* = 0.01). The absence of pulmonary artery dilation was also significantly linked to survival (*P* = 0.02), as was the absence of portal hypertension (*P* = 0.02). In contrast, the medication itself did not have a significant impact on survival (Table 2).

**Table 2:** Factors associated with mortality in Sch-PAH patients.

Factors	Death (n = 7)		Survival (n = 34)		*P*
	Mean/ N	SD/ %	Mean/ N	SD/ %	
Age	49.1	15	50.7	11.6	0.81^**†**^
Male	3	42.86	25	73.53	0.18*
Comorbidity	1	14.29	10	29.41	0.41*
*FC/NYHA*					
I/II	4	57.14	14	41.18	0.44*
III/IV	3	42.86	20	58.82	
6MWD(m)	262.9	43.1	303.5	87.1	0.08^**†**^
*Echo*					
PASP(mmHg)	81.3	30.9	86.1	23	0.73^**†**^
RA	6	85.71	33	97.06	0.31*
RV	6	85.71	33	97.06	0.31*
LA	2	28.57	16	47.06	0.36*
Pericardial effusion	1	14.29	3	8.82	0.54*
EF %	72.67	5.5	64.96	5.33	**0.01**^**†**^
*Heamodynamics*					
mRAP	12.5	6.06	9.39	5.94	0.29^**†**^
mPAP	55.5	12.8	54.8	11.8	0.91^**†**^
CI	2.265	0.885	2.57	1.09	0.48^**†**^
PVR	13.11	7.94	11.67	5.68	0.68^**†**^
Svo2	54.17	8.89	61.88	8.3	0.09^**†**^
PA dilatation	3	42.86	3	8.82	0.02*
PoHT	4	57.14	6	17.65	**0.02**
*Treatment*					
Selected medication	6	85.71	31	91.18	0.54*
Mono-Therapy	0	0	5	14.71	0.56*
Combination Therapy	6	85.71	26	76.47	0.59*
Diuretics	3	42.86	25	73.53	0.11*

Continuous data represented as mean and stander deviation (SD), and categorical data as number and percentage (%). N, number; SD, stander deviation; †: Independent t-test, *: Chi square test, *P* **< 0.05 **is statistically significant

FC, Functional class; NYHA, New York Heart Association; PA, Pulmonary artery; RA, right atrium; RV, right ventricle; LA, left atrium; EF, ejection fraction; 6MWD, 6 min’ walk distance.

mPAP, mean pulmonary artery pressure; mRAP, mean right atrial pressure; CI, cardiac index; PVR, pulmonary vascular resistance; SVo2, oxygen saturation in the mixed venous blood.

CT, computed tomography; PoHT, Porto Pulmonary hypertension.

Logistic regression analysis, both with and without adjustments for age, sex, and comorbidity, demonstrated that EF (%) served as an independent predictor of survival for these patients, with odds ratios (OR) of 0.72 and 0.55; *P* = 0.002 and < 0.001, respectively (Table 3).

**Table 3:** Predictors of mortality in Sch-PAH patients.

Factors	OR	95% CI	*P*
*un-adjusted*^*a*^			
EF %	0.72	(0.5516,0.9344)	**0.002**
*Adjusted*^*b*^			
EF%	0.55	(0.3181,0.9363)	** < 0.001**
Age	0.90	(0.7709,1.0557)	0.11
Male sex	4.11	(0.0832,203.4786)	0.44
Comorbidity	12.03	(0.1320,1095.7178)	0.21

OR, odd ratio; CI, confidence interval; a: Test of Goodness of fit = Hosmer–Lemeshow, X^2^ = 4.81, *P* = 0.55, b: Test of Goodness of fit = Hosmer–Lemeshow, X^2^ = 8.18, *P* = 0.42, P considered significant if < 0.05. Sch- PAH, Schistosomiasis-associated pulmonary arterial hypertension; EF, Ejection Fraction.

### Prognostic utility of left ventricle EF (%) measurement in patients with Sch-PHT

**Fig. 2:**
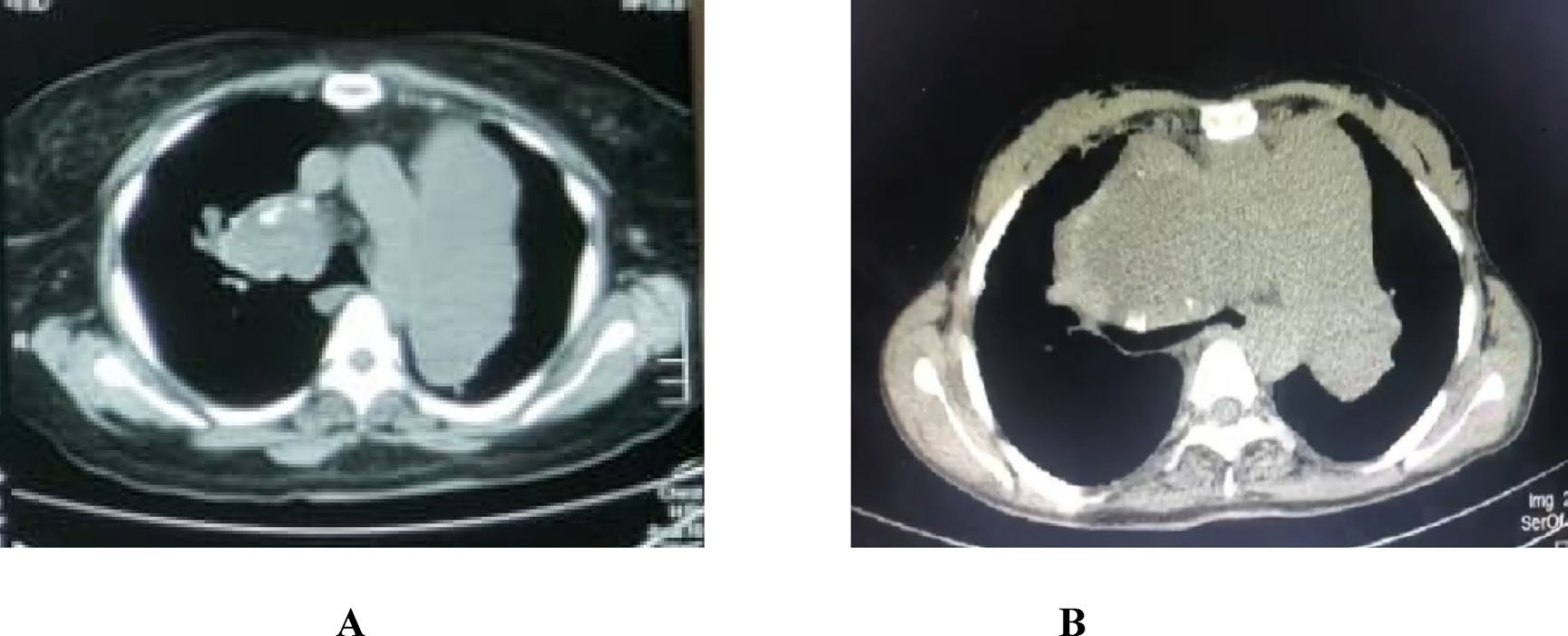
CT chest showed Pulmonary artery aneurysmal dilatation in 2 cases with Sch-PAH (**A** and **B**). Sch- PAH: Schistosomiasis-associated pulmonary arterial hypertension.

The use of ejection fraction (EF%) as a predictor of survival in patients with schistosomiasis-associated pulmonary arterial hypertension (Sch-PAH) was found to be highly informative, with an area under the curve (AUC) of 0.85 and a *p*-value of 0.008 (Fig. 2). At a cutoff value of 66%, the specificity was 100%, while the positive predictive value (PPV) and negative predictive value (NPV) were approximately 100% and 91%, respectively (Table 4).

**Table 4:** Prognostic performance of EF in patients with Sch-PHT.

Cutoff <	Sensitivity	95% CI	Specificity	95% CI	PV +	PV -
66	54%	0.3282 to 0.7445	100%	0.5407 to 1.000	100%	91%

CI, confidence interval; PPV, Positive predictive value; NPV, Negative predictive value.

Sch- PAH, Schistosomiasis-associated pulmonary arterial hypertension; EF, Ejection Fraction.

Among the patients with Sch-PAH, mortality data indicated that 5 out of 7 (71.4%) had a diagnosis of heart failure, while 2 out of 7 (28.6%) succumbed to liver cell failure.

## Discussion

In Egypt, schistosomiasis has historically been a major health issue. However, since the 1980s, the country has implemented several strategies to eradicate Schistosoma infestations, resulting in a dramatic decline in the number of cases over the past few decades^15^**.** The prevalence of *S. haematobium* and *S. mansoni* decreased from 15 and 40% in 1982 to 7% and 4% in 2000, respectively^16^**.**

Sch-PAH is a serious complication of chronic schistosomiasis infection and is a leading cause of morbidity and mortality related to PAH worldwide. The lack of understanding of the pathogenic mechanisms contributes to the absence of effective therapies, leading to worsening of the inflammatory condition and overall poor prognosis^17,18^.

Symptoms may manifest more than 20 years after the initial infection^12^, and typically occur during the third to fifth decades of life^19^. In the current study, the average age of Sch-PAH patients was found to be 50.4 years (SD ± 12), significantly higher than the average age of 34.5 years (± 9.7) in IPAH patients (*P* < 0.001). Notably, only one patient with Sch-PAH was under 30 years of age.

In contrast, Latin America exhibits a different age distribution, with two peaks of pulmonary hypertension observed in schistosomiasis patients: a younger peak and an older peak after the third decade^20^. Piscoya Roncal et al. reported in a retrospective cohort study conducted from 2004 to 2010, which included 68 patients from Socorro Cardiologica de Pernambuco, Brazil, that the mean age of patients was 46.6 years (± 12.9), with a female representation of 69.1% due to their continuous contact with contaminated water^21^.

Conversely, the current study revealed that the male percentage in the Sch-PAH group was 68.29%, likely due to increased exposure to contaminated freshwater in endemic areas through activities such as swimming, washing, and farming^22^. Patients with Sch-PAH tended to be classified in WHO functional classes II and III, whereas most patients from both groups were categorized as class IV. Based on the findings of Roncal et al. (2019), the majority of patients had functional classes III and IV (41 of 68)^21^.

This trend can be attributed to the older age of presentation among Sch-PAH patients and their associated comorbidities, which were statistically significant (*P* = 0.002) (Table 1). Consequently, older patients are more likely to be diagnosed at a more advanced stage than younger patients^23–25^. In contrast, IPAH is generally more prevalent among younger women^26,27^, which may motivate them to seek medical advice promptly upon experiencing health disruptions.

A combination of fatigue and aging can mask the warning symptoms of PAH, leading to delays in seeking medical care and a subsequent delay in diagnosis^23^(Table 1). This challenge was evident in the baseline risk assessment, where a greater proportion of IPAH patients were classified in the low-risk group (*P* = 0.01), while the Sch-PAH group exhibited the highest mortality risk (31.17% vs. 11.9%; *P* = 0.02) (Fig. 1).

Some patients with Sch-PAH exhibited dilated left atria, which is considered a marker for a subclinical degree of left heart involvement^28,29^. At the time of diagnosis, these changes did not result in pulmonary hypertension; however, they should be evaluated for their potential impact on future disease progression and cardiovascular comorbidities^29^. As indicated in Table 1, cardiovascular comorbidities were more prevalent in Sch-PAH patients than in those with IPAH; however, this difference did not reach statistical significance.

In contrast, Lapa et al. estimated the prevalence of PH to be 7.7% among patients diagnosed with hepatosplenic *S. mansoni* schistosomiasis, with a specific prevalence of precapillary PAH at 4.6%. They found that 3.0% of patients had postcapillary pulmonary hypertension^30^.

Right heart catheterization is essential to confirm the presence of elevated mPAP and to distinguish between precapillary and postcapillary types, as Sch-PAH patients may exhibit features of either or both^30,31^.

This study found no significant differences in hemodynamic profiles between patients with Sch-PAH and those with IPAH. These findings align with those of Fernandes et al. and Mendes et al.^32,33^, but in contrast to Hoette et al., the Sch-PAH patients exhibited a less severe hemodynamic profile^34^.

Moreover, some Sch-PAH patients showed significant pulmonary artery dilation compared to the IPAH group, with a mean diameter of 6.12 ± 2.18 cm (Table 1). Notably, several patients displayed aneurysmal dilation. These results are consistent with Hoette et al. (2015), which reported a mean pulmonary artery^23,34^ diameter of 4.5 ± 1.8 cm as shown in (Fig. 2).

In 1932, S. Azmi Pasha observed pronounced dilatation of the pulmonary arteries in the autopsy of two cases of pulmonary hypertension associated with hepatosplenic schistosomiasis^35^. Similarly, Hoette et al. stated that significant pulmonary artery enlargement is a characteristic radiological feature of schistosomiasis-associated pulmonary arterial hypertension (Sch-PAH) independent of the mean pulmonary artery pressure (mPAP) level, in contrast to idiopathic pulmonary arterial hypertension (IPAH)^34^.

The underlying reasons for this finding remain unclear. It is suggested that the pulmonary artery’s intrinsic properties may represent an adaptive response aimed at enhancing vascular capacity, thereby improving hemodynamics and reducing right ventricular load to preserve function^34^. Boerrigter et al. proposed that pulmonary artery dilatation might correlate more with the duration of the disease rather than the severity of the hemodynamic profile in PAH^36^.

Significant pulmonary artery dilatation may indicate the harmful impact of pulmonary hypertension on the vascular walls, contributing to more severe disease (Table 2). This is reflected by the fact that 42.86% of Sch-PAH patients who experienced death had pulmonary artery dilatation compared to only 8.82% among survivors.

In light of findings that indicate portal hypertension in only 26.8% of Sch-PAH cases, it appears that additional pathogenic pathways may contribute to pulmonary hypertension. For *S. mansoni*, some eggs remain trapped in the periportal venules of the hepatic portal system, leading to a localized immunological response that results in granuloma formation, pre-portal fibrosis, and chronic portal hypertension^37–40^. This process may facilitate the opening of portocaval shunts, allowing the migration of eggs from the portal system to the lungs^37–40^. Consequently, a severe immune response may occur, characterized by granuloma formation and type 2 inflammation mediated by IL-4 and IL-13, which can lead to remodeling of the pulmonary vessels^41–44^.

An alternative proposed mechanism involves the inflammatory cascade induced by Schistosoma eggs, rather than their burden, which results in diffuse vasculopathy known as obliterative arteritis^20^. Graham et al. (2011) noted that Schistosoma mansoni eggs or soluble egg antigens might not be present in the lungs of patients who died from pulmonary hypertension associated with chronic schistosomiasis^44^. Therefore, the prevailing notion is that an inflammatory cascade triggered by the egg antigen results in granuloma formation, contributing to the vascular disease, whether or not the granulomas are detected^19^.

While the prognostic data for schistosomiasis-associated pulmonary arterial hypertension (Sch-PAH) have not been extensively studied, preliminary findings suggest that Sch-PAH patients have a survival rate comparable to that of idiopathic pulmonary arterial hypertension (IPAH) patients^32^. It is also likely that their prognosis is similar to other forms of PAH, including IPAH^45^.

In terms of mortality during the study period, 7 out of 41 Sch-PAH patients (17.7%) died compared to 5 out of 42 IPAH patients (11.9%). This difference was not statistically significant, especially considering that Sch-PAH patients were generally older and had more frequent comorbidities.

This finding may contrast with other reports that assert a less severe baseline hemodynamic profile in Sch-PAH compared to IPAH, and that Sch-PAH patients often exhibit a slower progression of disease and improved survival^32^. Notably, one study indicated that the diagnosis of Sch-PAH, compared to IPAH, had a non-significant slight increase in the hazard ratio for death of 1.16^46^. Conversely, Knafl et al. (2020) suggested that Schistosoma-associated pulmonary arterial hypertension demonstrates better survival than idiopathic pulmonary arterial hypertension, although they did not provide data regarding the initial risk stratification of these patients^47^.

Despite previous registries indicating that PAH mortality is associated with male sex^48–50^, our study found no significant difference in mortality based on gender.

**Fig. 3:**
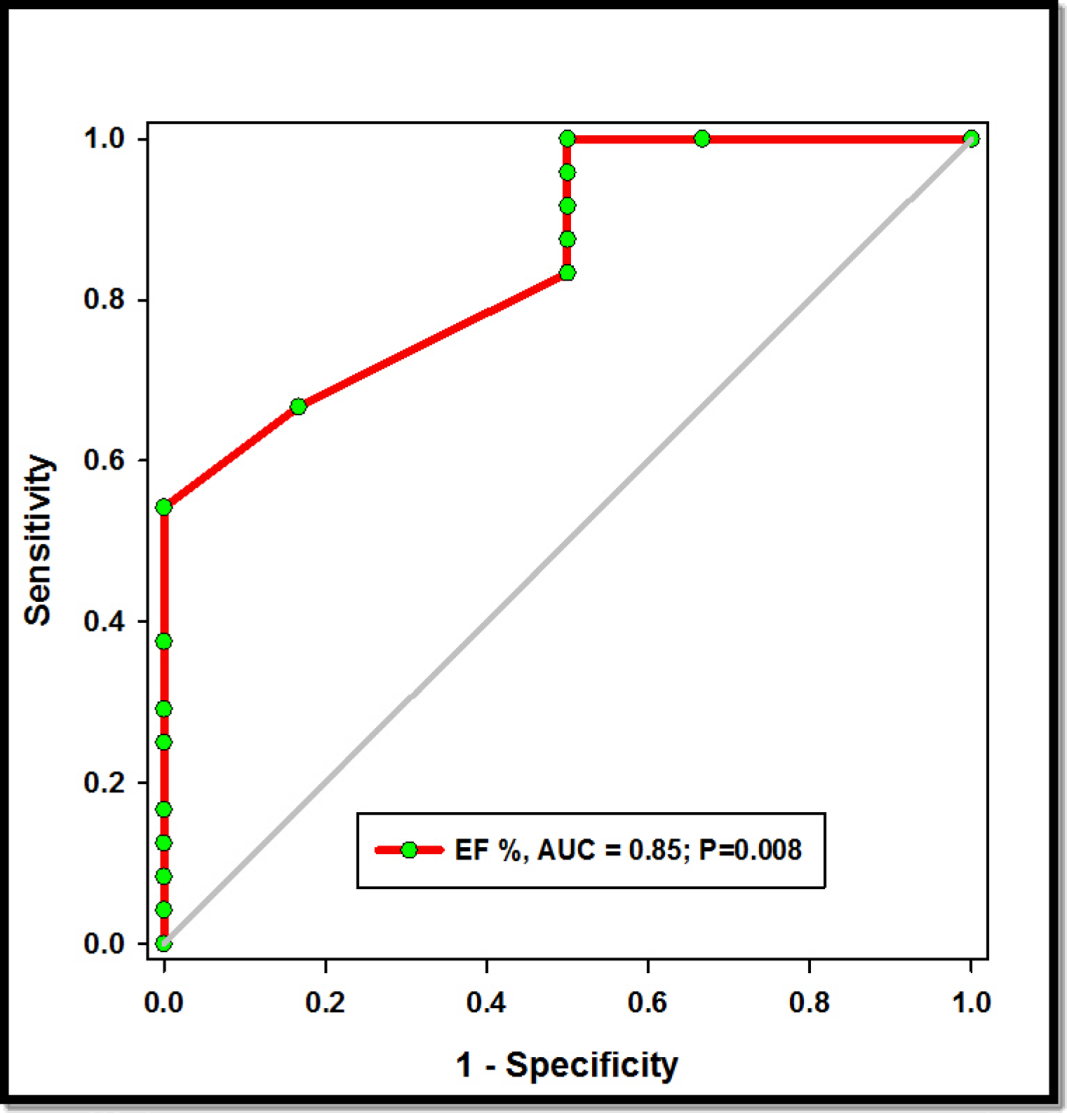
ROC curve of EF% for Sch-PHT prognosis. AUC: area under the curve, *P* < 0.05 considered significant. EF: Ejection Fraction, Sch- PAH: Schistosomiasis-associated pulmonary arterial hypertension.

Interestingly, this study highlighted that an ejection fraction greater than 66% is a critical determinant associated with mortality in Sch-PAH patients, showing a specificity of 100% (Tables 3, 4) (Fig. 3). This finding adds significant value to established prognostic variables in Sch-PAH. It should be noted that Sch-PAH group is older in age with high risk to develop diastolic heart failure than IPAP who are usually younger in age so this added worse prognosis as adding factor in addition to pulmonary hypertension and this could explain the cut value below 66% EF as predictor of mortality.

In cases of portal hypertension, Sch-PAH is associated with a hyperdynamic circulatory state and high cardiac output^12,41,51^. Badawya et al. observed that patients with schistosomiasis had elevated mean values for left ventricular ejection fraction (LVEF), right ventricular diameters, right atrial area, vena cava width, and pulmonary artery pressures. Conversely, these patients exhibited lower mean values for right ventricular ejection fraction (RVEF)^52^. High LVEF does not mean good prognosis as portal hypertension has associated hyperdynamic circulation and that increases cardiac output and with increase in pulmonary artery pressure that will cause bowing or shifting of interventricular septum towards the left side of the heart so EF of left side will increase gradually due to decrease in left ventricular end diastolic volume and this means decrease in capacity in left ventricle rather than improvement in contractility.

This may help explain the increased mortality observed in patients with a higher LVEF, as this could be indicative of greater impairment of RVEF. Additionally, they found that patients with coarse hepatic periportal fibrosis (PPF) displayed statistically significant lower values of RVEF and higher pulmonary artery pressures compared to those with fine PPF, which may contribute to our findings.

Anna Licata et al. reported that greater hyperdynamic circulation correlates with deterioration of hepatosplenic function, which may represent a preceding compensatory phase of end-stage liver disease, providing a potential link between higher mortality and increasing LVEF^53^.

The elevated trans-septal pressures from increased right ventricular pressure cause compression of the interventricular septum, leading to geometric and functional alterations in the left ventricle^17,18^. A previous study identified impairments in left ventricular contractility, even while conventional measures of left ventricular systolic function remained normal.

## Limitations

While this study offers valuable insights, there are several limitations to consider. First, the retrospective design may lead to less reliable data, making it challenging to establish cause and effect relationships. Although the sample size of 83 patients is reasonable, it may limit our ability to detect differences within smaller subgroups or rarer outcomes. Additionally, since this study was conducted in Egypt, the findings may not be generalizable to other countries with different experiences regarding schistosomiasis and pulmonary hypertension.

Our Schistosomiasis associated PAH group was less (41 patients in this study) as Egypt is now low endemic area of schistosomiasis with reduction of Schistosoma Mansoni and Haematobium prevalence in Egypt^15^ and only 5% of schistosomiasis could develop pulmonary arterial hypertension according to studies^47^ hence, patients of Sch associated PAH in our study were less.

Long-term follow-up data were not included, which would have been beneficial in understanding how Schistosomiasis-associated pulmonary hypertension (Sch-PAH) evolves over time and how various treatments may affect patient outcomes in the long run. Furthermore, reliance on echocardiographic measurements can introduce variability and may not be as precise, particularly in patients with multiple health issues. Thus, while the findings are noteworthy, these factors highlight the need to consider the broader context when interpreting the results.

There were drug limitations for specific pulmonary hypertension availability due to affordability in addition to availability in market so mainly we used Sildenafil, Bosentan or both as combination therapy and that was the reason for incapability of comparison of different specific pulmonary hypertension medications with outcomes.

## Conclusion

Sch-PAH primarily affects older male patients, who frequently present with significant comorbidities and more severe clinical manifestations compared to those with IPAH. Given the elevated prevalence of schistosomiasis, particularly in endemic regions, Sch-PAH represents a critical public health challenge. Identifying key prognostic factors, such as left atrial dilation, pulmonary artery dilation, and ejection fraction levels above 66%, is essential for early recognition of patients at high risk for poor outcomes. These predictors can significantly enhance patient management strategies and improve survival rates among individuals suffering from Sch-PAH.

## Data Availability

All data are included in the tables.
